# Outcomes of notifications to health practitioner boards: a retrospective cohort study

**DOI:** 10.1186/s12916-016-0748-6

**Published:** 2016-12-02

**Authors:** Matthew J. Spittal, David M. Studdert, Ron Paterson, Marie M. Bismark

**Affiliations:** 1 Melbourne School of Population and Global Health, The University of Melbourne, Parkville, Victoria 3010 Australia; 2 Stanford University School of Medicine and Stanford Law School, 117 Encina Commons, Stanford, CA 94305 USA; 3 Auckland Law School, The University of Auckland, Private Bay 92019, Auckland, 1142 New Zealand; 4 Melbourne Law School, The University of Melbourne, Parkville, Victoria 3010 Australia

**Keywords:** Disciplinary action, Doctors, Nurses, Dentists, Pharmacists, Psychologists

## Abstract

**Background:**

Medical boards and other practitioner boards aim to protect the public from unsafe practice. Previous research has examined disciplinary actions against doctors, but other professions (e.g., nurses and midwives, dentists, psychologists, pharmacists) remain understudied. We sought to describe the outcomes of notifications of concern regarding the health, performance, and conduct of health practitioners from ten professions in Australia and to identify factors associated with the imposition of restrictive actions.

**Methods:**

We conducted a retrospective cohort study of all notifications lodged with the Australian Health Practitioner Regulation Agency over 24 months. Notifications were followed for 30–54 months. Our main outcome was restrictive actions, defined as decisions that imposed undertakings, conditions, or suspension or cancellation of registration.

**Results:**

There were 8307 notifications. The notification rate was highest among doctors (IR = 14.5 per 1000 practitioners per year) and dentists (IR = 20.7) and lowest among nurses and midwives (IR = 2.0). One in ten notifications resulted in restrictive action; fewer than one in 300 notifications resulted in suspension or cancellation of registration. Compared with notifications about clinical care, the odds of restrictive action were higher for notifications relating to health impairments (drug misuse, OR = 7.0; alcohol misuse, OR = 4.6; mental illness, OR = 4.1, physical or cognitive illness, OR = 3.7), unlawful prescribing or use of medications (OR = 2.1) and violation of sexual boundaries (OR = 1.7). The odds were higher where the report was made by another health practitioner (OR = 2.9) or employer (OR = 6.9) rather than a patient or relative. Nurses and midwives (OR = 1.8), psychologists (OR = 4.5), dentists (OR = 4.7), and other health practitioners (OR = 5.3) all had greater odds of being subject to restrictive actions than doctors.

**Conclusions:**

Restrictive actions are the strongest measures health practitioner boards can take to protect the public from harm and these actions can have profound effects on the livelihood, reputations and well-being of practitioners. In Australia, restrictive actions are rarely imposed and there is variation in their use depending on the source of the notification, the type of issue involved, and the profession of the practitioner.

## Background

Many countries entrust oversight of doctors and other health professionals to practitioner boards. A core mission of such boards is to protect the public from unsafe practice. Boards rely on patients, practitioners and their peers, employers and other agencies to bring risks to their attention and can employ a range of assessment and investigation processes to evaluate concerns about a practitioner’s health, performance or conduct. In cases where a practitioner poses a risk to patient safety boards can initiate a range of actions, including imposing conditions on the practitioner’s registration or suspending their licensure to practice. Actions that restrict ability to practice may have profound effects on affected practitioners – damaging livelihood, reputation, and potentially personal well-being [1, 2]. Imposing such actions therefore requires boards to chart a delicate course between protecting patients from harm and respecting the rights of practitioners [3].

Previous research has examined factors associated with disciplinary action against doctors. Studies have compared disciplined doctors with controls drawn from the broader medical workforce [4–7], with colleagues who were investigated or charged but not disciplined [8], and with colleagues who incurred less serious sanctions [9]. In general, these studies identify several risk factors for incurring disciplinary sanctions, including male sex, late career stage, and practice in certain specialties (surgery, obstetrics and gynecology, psychiatry, and general practice). In addition, longitudinal studies of doctors have shown higher rates of disciplinary actions among physicians who performed poorly during residency [10] and physicians who lack specialty certification [11]. Relatively few studies have focused these types of analyses on nurses [12–14], and fewer still have examined pharmacists [15], psychologists [16], dentists [17, 18], and other allied health professions. Moreover, this body of research tends to be profession specific. For example, no previous studies have directly compared doctors’ likelihood of regulatory action with risks experienced by other health practitioners.

In Australia, 14 health professions, including doctors, nurses, dentists, psychologists, and pharmacists, are regulated by a unified scheme that has operated since 2010. The scheme covers all states and territories, which creates a rare opportunity to use national, longitudinal data to examine the incidence and outcomes of “notifications of concern” (hereafter, “notifications”) relating to multiple professions [19]. We conducted a retrospective cohort study of all notifications received by the national agency over a 2 year period. We estimated the incidence of notifications among health practitioners and tested for associations between various characteristics of notified practitioners (e.g., age, sex, profession) and notifications (e.g., issue type and source of notification) and the adjudicated outcomes of these notifications, particularly restrictive actions.

Our goal was to advance understanding of how this key regulatory regime operates. We were particularly interested in generating information with the potential to facilitate efficient adjudication and guide prevention efforts. We hypothesized that there would be systematic differences in rates of notification between professions, and that there would be relatively high rates of restrictive action against practitioners from certain professions (e.g., psychologists) and for notifications about certain issues (e.g., drug or alcohol misuse).

## Methods

### Setting

Australia’s system for regulating health practitioners incorporates a nationally-consistent process for registration across 14 professions [20]. Notifications regarding the health, conduct, and performance of practitioners are also consistently managed under the National Registration and Accreditation Scheme (Table 1), apart from two jurisdictions who operate alternative models of co-regulation: New South Wales (since the establishment of the scheme in July 2010) and Queensland (since July 2014) [19]. Ten professions have been regulated by the scheme since inception (medical practitioners, nurses and midwives, dental practitioners, psychologists, pharmacists, chiropractors, optometrists, osteopaths, physiotherapists, and podiatrists). A further four joined in July 2012 (Aboriginal and Torres-Strait Islander practitioners, Chinese medicine practitioners, medical radiation practitioners, and occupational therapists). Most notifications are made voluntarily by an individual or organization who wishes to raise a concern about a health practitioner. Mandatory notification by a fellow practitioner or employer is required in certain situations, such as where a practitioner has practiced while intoxicated by alcohol or drugs. Notifications are lodged with the Australian Health Practitioner Regulation Agency (AHPRA), before being referred to the relevant national board (e.g., the Medical Board of Australia, the Nursing and Midwifery Board of Australia).

**Table 1:** Overview of notifications under the National Registration and Accreditation Scheme

Who can be subject to a notification?	All registered health practitioners from 14 health professions^a^
Who can make a notification?	Patients or relatives, self, fellow practitioners, employers, agencies, members of the public, complaint commissioners
When can action be taken against a health practitioner? (ss 178, 191, 196)	A Board may take action if a practitioner:
	• Has behaved in a way that is unsatisfactory
	• Practices in a way that is unsatisfactory
	• Has or may have an impairment
	A Panel or Tribunal may take action if a practitioner:
	• Has behaved in a way that constitutes unsatisfactory professional performance
	• Has behaved in a way that constitutes unprofessional conduct or professional misconduct
	• Has an impairment
What actions may be taken? (ss 178, 191, 196)	Non-restrictive actions
	• No further action
	• Referral to another body
	• Caution, reprimand or fine
	Restrictive actions
	• Undertaking from the practitioner
	• Imposition of condition on registration
	• Suspension or cancellation of registration

^a^Our study excluded four professions that joined the national scheme late

With support from AHPRA, boards assess each notification and then initiate a more in-depth investigation in cases where this appears necessary. A board may decide no further action is warranted (before or after an investigation), require the practitioner to undergo an assessment of their health or performance, refer the matter to another regulatory body, or take the matter to a hearing before a tribunal.

Final outcomes take several different forms. Non-restrictive actions – typically a caution, reprimand or fine – may have financial or reputational consequences for practitioners but do not restrict their registration in any way. Restrictive actions are those which limit the practice of the profession or require practitioners to do certain things. The restrictive actions available are accepting an undertaking from the practitioner related to their clinical practice (e.g., further training, random drug testing, counselling); imposing specified conditions on practice (e.g., have a chaperone present when seeing female patients); suspending registration for a specified period; and seeking to cancel the practitioner’s registration. In some cases, the final decision will be made by a disciplinary tribunal rather than the board itself and only tribunals have the power to cancel a practitioner’s registration. A guiding principle of the scheme is that restrictive actions should involve the minimum regulatory force appropriate to manage the risk.

### Study design

Using administrative data routinely collected by AHPRA, we identified all notifications about the health, performance, or conduct of a health practitioner lodged in 2011 and 2012. We then followed these notifications for 30–54 months (through to 30 June 2015) to identify their outcomes. We used data from the register of health practitioners to calculate notification rates and to identify predictors of restrictive actions.

### Data collection

AHPRA provided us with data on all health practitioners registered between 1 January 2011 and 31 December 2012. This “practitioner extract” consisted of variables indicating the period during which each practitioner was registered; the practitioner’s age band, sex, profession, and state or territory of practice; and the remoteness, based on the practice location provided by the practitioner [21].

AHPRA also provided a data extract relating to all notifications lodged about registered practitioners during the same 2-year period. This “notification extract” included information collected at the time the notification was lodged (e.g., lodgment date, source of notification, primary issue raised), as well as information relating to the ensuing adjudication (e.g., closure date, case outcome). Anonymized, unique identifiers enabled us link the practitioner extract to the notification extract.

Data on notifications were not available from New South Wales (*n* = 188,297 practitioners). Although health practitioners in New South Wales are subject to similar requirements as those in other states, they are managed through separate co-regulatory arrangements. This means AHPRA does not hold the same kind of detailed information about the management of these notifications as it does for notifications from other jurisdictions. We also excluded practitioners in the four health professions that joined the scheme in 2012 (*n* = 35,954) and practitioners registered to an address outside Australia (*n* = 14,576). The notifications of interest thus came from all the other practitioners who were registered during 2011 and 2012 (*n* = 349,480).

### Measures

To protect confidentiality, AHPRA provided practitioners’ birth dates in 5-year bands (e.g., 1970–1974). We recoded this variable to reflect each practitioner’s age group in 2010. We coded health professions into six categories: doctors, nurses and midwives, psychologists, pharmacists, dentists, and other health practitioners (chiropractors, optometrists, osteopaths, physiotherapists, podiatrists, oral health therapists, dental hygienists, dental prosthetics, and dental auxiliaries). The primary issue involved in each notification was originally coded into one of 149 categories. We recoded these issues into four categories and 15 sub-categories. These were performance issues (concerns about clinical care, poor communication, concerns about medication, delays in access to care); conduct issues (disruptive behavior, improper use or management of health information, non-compliance with regulatory requirements, unlawful use or supply of medications, unfair costs or misleading advertising, breaches of boundaries); health impairment issues (mental illness, drug misuse, alcohol misuse, physical or cognitive illness); and other issues.

The register of practitioners changes daily. We therefore used data on the dates practitioners became registered and unregistered with AHPRA to calculate practitioners’ exposure time – the period each practitioner could potentially receive a notification. For most practitioners, their exposure time began on 1 January 2011 and ended on 31 December 2012 (when observation of notifications ceased). For practitioners whose registration began and/or ended within this interval (e.g., new graduates, retirees), their exposure time was adjusted accordingly.

### Analyses

We used counts and percentages to describe the characteristics of notifications, including the reporting source, the primary issue, the time taken to resolve each notification, and the final determination. We then conducted three separate analyses.

First, we used negative binomial regression to estimate the incidence of notifications by practitioner profession, age, sex, area remoteness, and jurisdiction. By computing marginal effects, derived directly from model estimates [22, 23], we are able to report these findings as adjusted incidence rates. We then performed multivariate logistic regression analysis to examine the factors associated with restrictive actions (undertaking, imposition of conditions, suspension or cancellation of registration) and a sensitivity analysis examining factors associated with any regulatory action (restrictive actions, cautions, reprimands or fines).

All analyses were conducted using Stata 13.1 [24].

## Results

### Notification rates

In 2011–2012, 8307 notifications pertaining to 6920 practitioners were lodged with AHPRA. The overall rate was 6.3 notifications per 1000 practitioners per year (95% CI, 6.2 to 6.5).

Notification rates differed by profession, age, sex, and jurisdiction (Table 2). After adjusting for all of the variables shown in Table 2 plus jurisdiction, dentists had the highest rate of notifications (20.7 per 1000 practitioners per year), followed by doctors (14.5 per 1000 practitioners per year). Nurses and midwives had the lowest rate of notifications (2.0 per 1000 practitioners per year). Risk of notification generally increased with age – practitioners aged ≤ 25 years were at lowest risk (2.6 per 1000 practitioners per year) and practitioners aged 56–65 years were at highest risk (8.5 per 1000 practitioners per year). Men were at much higher risk of notification than women (8.9 vs. 4.0 per 1000 practitioners per year). Notification rates did not differ by remoteness of practice location (*P* = 0.48), but did by jurisdiction (*P* < 0.0001).

**Table 2:** Number of notifications and adjusted notification rate per 1,000 practitioners per year

Characteristic	Number of notifications n = 8307^a^	Adjusted notification rate per 1000 practitioners per year^b^	95% confidence interval	*P* value^c^
Profession				<0.0001
Doctor	4504	14.5	13.9 to 15.1	
Nurse and/or midwife	1537	2.0	1.9 to 2.1	
Psychologist	473	7.1	6.4 to 7.7	
Pharmacist	409	6.8	6.1 to 7.5	
Dentist	910	20.7	18.9 to 22.5	
Other health practitioner	474	4.5	4.1 to 5.0	
Age in 2010				< 0.0001
≤ 25	255	2.6	2.3 to 3.0	
26–35	1334	4.0	3.8 to 4.2	
36–45	2104	6.4	6.1 to 6.8	
46–55	2594	8.2	7.8 to 8.6	
56–65	1582	8.5	8.0 to 9.0	
≥ 66	438	8.2	7.2 to 9.2	
Sex				< 0.0001
Female	2938	4.0	3.8 to 4.1	
Male	5367	8.9	8.6 to 9.2	
Practice location				0.48
Major cities	6343	6.2	6.1 to 6.4	
Inner/outer regional	1840	6.0	5.7 to 6.3	
Remote/very remote	117	5.9	4.6 to 7.2	

^a^Some cells do not sum to 8307 notifications because of missing data

^b^Adjusted for all other variables in the table and state/territory

^c^ *P* value refers to evidence that the adjusted notification rates differs between categories. This test is based on the coefficients (and their standard errors) from the negative binomial model

### Source and type notifications

Nearly one-third of the notifications were lodged by patients or relatives and another 30% were lodged by state complaint commissioners (Table 3). The next most common sources of notifications were fellow practitioners (13.0%) and employers (9.5%).

**Table 3:** Source and issue of notifications

	Notifications N = 8307 Number (%)
Source	
Patient or relative	2741 (33.0)
Complaint commissioner	2508 (30.2)
Fellow practitioner	1079 (13.0)
Employer	789 (9.5)
Other agency	441 (5.3)
Self	202 (2.4)
Unknown	547 (6.6)
Issue	
Performance^a^	3197 (38.5)
Concerns about clinical care	2550 (30.7)
Poor communication	368 (4.4)
Concerns about medication	145 (1.8)
Delays in access to care	134 (1.6)
Conduct^a^	2616 (31.5)
Disruptive behavior	672 (8.1)
Improper use or management of health information	628 (7.6)
Non-compliance (admin/regulatory requirements/fraud)	382 (4.6)
Unlawful use or supply of medications	364 (4.4)
Unfair costs or misleading advertising	349 (4.2)
Breaches of boundaries	221 (2.7)
Health^a^	462 (5.6)
Mental illness	202 (2.4)
Drug misuse	116 (1.4)
Alcohol misuse	81 (1.0)
Physical/cognitive illness	63 (0.8)
Other issue^a^	572 (6.9)
Unknown	1460 (17.6)

^a^category heading (subcategories underneath)

Thirty-eight percent of notifications involved concerns about performance, 31.5% involved concerns about conduct, and 5.6% involved concerns about the health of practitioners. Among notifications about performance, the most common issues arising were concerns about the quality of clinical care (30.7%) and communication (4.4%). Among notifications about conduct, allegations of disruptive behavior were the most common (8.1%), followed by concerns about improper use or management of health information (7.6%) and non-compliance with regulatory or administrative requirements (4.6%). Alleged breaches of sexual boundaries accounted for 2.7% of notifications.

### Outcomes of notifications

Approximately 36% of notifications were resolved within 3 months, 74% within 1 year and 91% within 2 years. By the end of our follow-up period, 7898 (95%) of the notifications in the study sample had reached a final determination. The remaining results relate to these “closed” notifications.

**Fig. 1:**
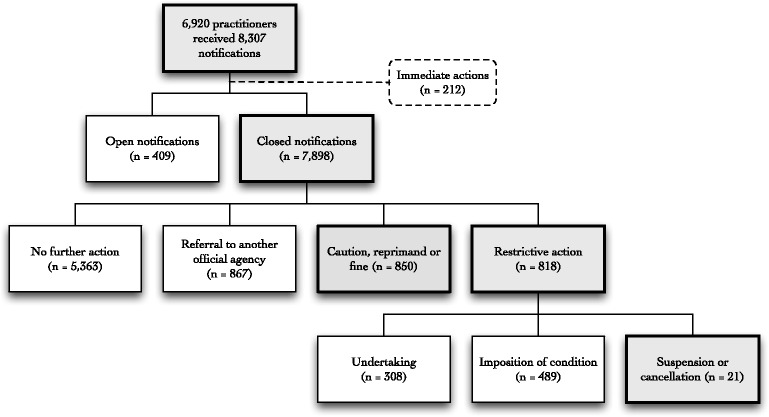
Overview of the number of practitioners, number of notifications, and outcome of notifications

For 68% (5363/7898) of closed notifications, the final determination was to take no further action after the Board had considered the issues raised (Fig. 1) (a decision to take no further action does not necessarily mean that the notification was groundless: it may mean that the threshold for regulatory action was not met or that there is no longer a risk to the public that needs to be managed because of actions the practitioner took during the assessment or investigation period). Ten percent (818/7898) of closed notifications resulted in restrictive actions, 11% (850/7898) in a caution, reprimand or fine, and 11% (867/7898) were referred to another official agency (e.g., police).

Among notifications ending in restrictive actions, 38% (308/818) involved practitioners entering a legally enforceable but voluntary undertaking with the board; 60% (489/818) involved the imposition of formal conditions on the practitioner’s registration; and 3% (21/818) involved removal from practice through temporary suspension or permanent cancellation of registration by a tribunal. The 21 removals related to 19 different practitioners, and 14 of these notifications involved conduct issues (seven were breaches of boundaries), four involved performance issues, and one involved a health issue.

### Predictors of restrictive action

The source of the notification, the primary issue of concern in the notification, and the notified practitioner’s profession were all strongly associated with the odds of a restrictive action (Table 4, columns 2 and 3).

**Table 4:** Multivariate predictors of restrictive actions (undertaking, imposition of conditions, suspension or cancellation of registration) and any regulatory action (restrictive actions plus cautions, reprimands or fines)^a^

	Restrictive actions		Any regulatory action	
Characteristic	Odds ratio (95% CI)	*P* value	Odds ratio (95% CI)	*P* value
Source of notification		< 0.0001		< 0.0001
Patient or relative (ref.)	1.0		1.0	
Complaints commissioner	1.0 (0.8 to 1.4)		1.1 (0.9 to 1.3)	
Fellow practitioner	2.9 (2.2 to 3.8)		1.9 (1.5 to 2.3)	
Employer	6.9 (5.1 to 9.3)		4.2 (3.3 to 5.3)	
Other agency	5.3 (3.8 to 7.4)		3.2 (2.5 to 4.1)	
Self	4.0 (2.6 to 6.3)		2.6 (1.8 to 3.8)	
Unknown	2.2 (1.5 to 3.3)		1.4 (1.0 to 1.9)	
Issue		< 0.0001		< 0.0001
Performance: Concerns about clinical care (ref.)	1.0		1.0	
Performance: Poor communication	0.5 (0.3 to 0.8)		0.7 (0.5 to 1.0)	
Performance: Concerns about medication	1.5 (0.8 to 2.8)		2.7 (1.8 to 3.9)	
Performance: Delayed access	0.2 (0.1 to 0.9)		0.5 (0.3 to 1.0)	
Conduct: Disruptive behavior	0.8 (0.6 to 1.1)		0.8 (0.6 to 1.0)	
Conduct: Improper use or management of health information	0.6 (0.4 to 0.9)		1.0 (0.8 to 1.3)	
Conduct: Breaches of boundaries	1.7 (1.1 to 2.5)		1.8 (1.3 to 2.6)	
Conduct: Non-compliance (admin/regulatory requirements/fraud)	0.5 (0.4 to 0.8)		0.7 (0.5 to 0.9)	
Conduct: Unlawful use or supply of medications	2.1 (1.4 to 3.0)		2.7 (2.0 to 3.6)	
Conduct: Unfair costs or misleading advertising	0.1 (0.0 to 0.2)		0.2 (0.1 to 0.3)	
Health: Mental illness	4.1 (2.8 to 6.1)		2.0 (1.4 to 2.8)	
Health: Drug misuse	7.0 (4.4 to 11.2)		3.4 (2.2 to 5.2)	
Health: Alcohol misuse	4.6 (2.8 to 7.7)		2.5 (1.5 to 4.1)	
Health: Physical/cognitive illness	3.7 (2.0 to 6.8)		1.7 (1.0 to 3.0)	
Other issues	1.0 (0.7 to 1.3)		1.0 (0.8 to 1.3)	
Profession		< 0.0001		< 0.0001
Doctor (ref.)	1.0		1.0	
Nurse and/or midwife	1.8 (1.4 to 2.4)		1.5 (1.3 to 1.9)	
Psychologist	4.5 (3.2 to 6.3)		2.6 (2.0 to 3.4)	
Pharmacist	0.8 (0.5 to 1.4)		3.1 (2.3 to 4.1)	
Dentist	4.7 (3.5 to 6.4)		2.4 (1.9 to 3.0)	
Other health practitioner	5.3 (3.8 to 7.4)		2.8 (2.1 to 3.6)	
Age in 2010		0.029		0.83
≤ 25	0.9 (0.6 to 1.5)		1.1 (0.7 to 1.5)	
26–35	1.0 (0.8 to 1.3)		1.1 (0.9 to 1.3)	
36–45	1.0 (0.8 to 1.3)		1.1 (0.9 to 1.3)	
46–55 (ref.)	1.0		1.0	
56-65	1.2 (0.9 to 1.6)		1.1 (0.9 to 1.3)	
≥ 66	1.9 (1.3 to 2.8)		1.2 (0.9 to 1.6)	
Sex		0.59		0.079
Female (ref.)	1.0		1.0	
Male	1.1 (0.9 to 1.3)		1.1 (1.0 to 1.3)	
Remoteness		0.35		0.42
Major cities (ref.)	1.0		1.0	
Inner/outer regional	1.2 (0.9 to 1.4)		1.1 (0.9 to 1.3)	
Remote/very remote	0.8 (0.4 to 1.7)		0.8 (0.5 to 1.4)	

^a^Models also adjusted for jurisdiction

Compared with concerns about clinical care (the reference category), the odds of a restrictive action being imposed were significantly higher among notifications involving health impairments (drug misuse OR = 7.0, alcohol misuse OR = 4.6, mental illness OR = 4.1, physical or cognitive illness OR = 3.7), unlawful use or supply of medications (OR = 2.1), and breach of boundaries (OR = 1.7). On the other hand, the odds of restrictive action were significantly lower among notifications involving unfair costs or misleading advertising (OR = 0.1), delayed access to care (OR = 0.2), non-compliance with administrative or regulatory requirements (OR = 0.5), poor communication (OR = 0.5), and problems with the use of health information (OR = 0.6).

Compared with notifications lodged by patients or relatives, the odds of restrictive action were significantly higher among notifications lodged by employers (OR = 6.9), other agencies such as a health department (OR = 5.3), and fellow practitioners (OR = 2.9). Self-notifications also had significantly higher odds of ending in restrictive actions (OR = 4.0); these arise in a variety of situations, for example, a practitioner may self-notify a substance abuse issue to pre-empt notification by a third party.

The odds of restrictive action being taken in relation to notifications against dentists (OR = 4.7) and psychologists (OR = 4.5) were more than four times higher than in notifications against doctors, and nearly two times higher than in notifications against nurses and midwives (OR = 1.8). Notifications against male practitioners were no more likely to end in restrictive actions than those against female practitioners, nor were there differences in the odds of restrictive action according to the remoteness of the notified practitioner’s practice location.

### Predictors of any regulatory action

The pattern of results when the outcome was any regulatory action (restrictive actions, cautions, reprimands, or fines) was broadly similar, although most ORs were smaller than those for restrictive actions alone (Table 4, columns 4 and 5). The notable difference was for pharmacists. In this analysis they had odds of regulatory action that were nearly three times that of doctors suggesting that, compared with doctors, pharmacists who were the subject of a notification were more likely to receive cautions, reprimands, or fines.

### Time to resolution

The time involved in resolving a case increased with the severity of the outcome. The median time to closure for cases that resulted in no further action was 9 months, compared with 34 months for cases that involved any regulatory action, 37 months for cases resulting in restrictive action, and 70 months for the 21 cases that resulted in suspension or cancellation of registration. Some of these delays fall outside of the control of AHPRA or the Boards, and relate, for example, to time spent waiting for a coroner or tribunal to conclude their consideration of a case.

## Discussion

Health practitioner boards are pivotal institutions in the regulation of healthcare delivery [25]. They play a critical role in deciding who is lawfully entitled to practice and in protecting the public from substandard practice. How practitioner boards execute their regulatory function has important consequences for affected practitioners [1], the profession, and the public [2].

### Main findings

This study of notifications lodged over a 2-year period against practitioners from 10 health professions found an overall rate of six notifications per 1000 practitioners per year. Doctors and dental practitioners had relatively high notification rates and nurses and midwives had relatively low rates. Final determinations were made on the majority of notifications within a year, although around one in ten took more than 2 years to resolve. In nearly 70% of cases, no further action was taken. About 10% of notifications resulted in restrictive actions, almost all of which involved some form of undertaking or conditions on practice. Only 21 notifications – about 0.3% of the total lodged – resulted in removal from practice. Notifications from peers and employers, notifications about health problems (particularly drug or alcohol problems), and notifications against dentists and psychologists had the highest odds of ending in restrictive actions.

### Strengths and limitations

An important strength of this study is the ability to analyze notifications from multiple jurisdictions and multiple professions, through the use of data from Australia’s National Registration and Accreditation Scheme. A few previous studies have shown inter-board variation in disciplinary decisions within a single profession [2, 26, 27], but none have compared decisions by boards regulating different professions. An additional strength of the study is its long follow-up time, which allowed us to observe the final outcomes of the vast majority of notifications.

The most significant limitation of the study is that we were not able to measure a number of practitioner-level variables that are likely to be related to the risks of regulatory action. These include patient volume, type of practice, history of disciplinary actions and, for doctors, performance issues during training and the country of their primary medical degree. Previous studies have identified associations between these factors and disciplinary outcomes [10, 12, 15, 28].

Second, despite the lengthy follow-up period, 5% of the notifications in our sample did not have final decisions at the time the study data were extracted. Cases that take longer to resolve tend to involve more serious outcomes. Findings of serious misconduct are typically made by a tribunal after a hearing, which occurs at the end of the adjudication process, and the tribunal’s decision in such cases may be appealed [29]. The implication of this for our findings is that we may underestimate the number of notifications that end in restrictive actions, especially those involving suspension or cancellation of practice.

Third, we note that the Australian national scheme was still in its early years at the time of this study. Differences in decision-making between professions may reduce over time as Boards work together to develop more consistent approaches to the assessment and resolution of notifications.

### Interpretation and implications

The regulation of health practitioners in Australia has the primary objective of protection of the public by ensuring that only competent and ethical practitioners are registered. Handling notifications of concern about a practitioner’s health, conduct or performance looms as a key role of the national multi-practitioner regulation agency, with a complex apparatus for receiving, assessing and investigating notifications. This study suggests some interesting lessons for other international health practitioner boards.

We found that notifications to practitioner boards about the health, conduct or performance of a health practitioner are a rare event, and responses to notifications that involve restrictive actions, such as conditions on or removal from practice, are rarer still.

The probability of restrictive actions varied widely depending on the source of the notification (with notifications from peers more likely to result in restrictive action than those made by patients) and by profession (with notifications about doctors less likely to result in restrictive actions than those against other professions).

The evidence that notifications made by fellow practitioners or employers are much more likely to lead to restrictive action than notifications made by patients and relatives or by complaint commissioners (which generally respond to patient complaints [30]) is perhaps unsurprising. One possible explanation is that peers are better positioned to recognize legitimate bases for a notification than are patients [2]. Relatedly, peers may refrain from notifying in all but the most egregious instances. A competing explanation relates to the adjudication process: notifications by peers may receive closer attention than notifications by patients. Given the increasing emphasis on the role of patients and families in patient safety, it would be of concern if issues raised by those on the receiving end of care are discounted in assessment processes dominated by peer opinion.

We found that nearly half of all notifications were due to concerns about performance issues, yet very few of these notifications resulted in restrictive actions. In contrast, relatively few notifications concerned health impairments, but a substantially larger proportion of them ended in restrictive actions. Part of the explanation for this difference may relate to issues of evidence and proof. What constitutes an unacceptable level of performance may be more difficult to determine, both for the notifier and the adjudicator, than the existence of an impairment that endangers safe care. Performance concerns inevitably raise the specter of judging what is acceptable care, an area where regulators (advised by members of the profession) have always treaded warily. Professional reticence to criticize poor care [31] and vigorous defense lawyers likely also play a part.

Consistent with previous studies, notification rates were higher among male practitioners and older practitioners [32]. However, there was no difference between males and females in the odds of restrictive action once a notification was received, and there was no trend for age.

Finally, the variation we observed between professions, both in the rate at which notifications were made, and the rate at which notifications ended in restrictive actions, is striking. Doctors, for example, had one of the highest rates of notifications, but those notifications were less likely to result in restrictive actions than in other professions. There are several possible explanations for such variation – they may be due to inter-professional differences, such as whether substandard care may directly cause harm (including pain), in the underlying rate of unprofessional behaviors, in the likelihood that any given episode of unprofessional behavior will be notified, in “case mix” (i.e., the nature and legitimacy of notifications), and in how strictly boards respond. We cannot disentangle these competing explanations because we did not observe unprofessional behavior directly, other than through the decisions of boards in cases that reach them.

## Conclusions

Health practitioner boards are charged with upholding professional standards and maintaining public confidence in regulated health professions. This requires taking timely and appropriate action in response to concerns about the health, conduct, or performance of health practitioners. Our findings reveal significant variation in the use of restrictive actions depending on the source of the notification, the type of issue involved, and the profession of the practitioner. Further work is required to understand whether such variations are to be expected based on the nature and magnitude of the risks involved, and whether restrictive actions are effective in protecting the public from a future risk of harm.
