# Correction to: Outcomes of notifications to health practitioner boards: a retrospective cohort study

**DOI:** 10.1186/s12916-018-1030-x

**Published:** 2018-03-07

**Authors:** Matthew J. Spittal, David M. Studdert, Ron Paterson, Marie M. Bismark

**Affiliations:** 1 Melbourne School of Population and Global Health, The University of Melbourne, Parkville, Australia; 2 Stanford University School of Medicine and Stanford Law School, Stanford, USA; 3 Auckland Law School, The University of Auckland, Auckland, New Zealand; 4 Melbourne Law School, The University of Melbourne, Parkville, Australia

## Erratum

The original article [1] contains a major error whereby all rates in Table 2 are mistakenly presented as 50% of their true values; this error was caused by a miscalculation in annualising the original values that represented the rates.

The correct version of Table 2 can be seen below whereby each rate is presented without having been divided by two, and each rate’s units are expressed as being ‘per 1000 practitioner years’ rather than ‘per 1000 practitioners per year’. This Table should be taken into account over the version of Table 2 seen in the original article [1].

**Table 2:** Number of notifications and adjusted notification rate per 1,000 practitioner years

Characteristic	Number of notifications *n* = 8,307 (a)	Adjusted notification rate per 1,000 practitioner years (b)	95% confidence interval	*p*-value (c)
Profession				< 0.0001
Doctor	4,504	29.0	27.8 to 3.2	
Nurse and/or midwife	1,537	4.1	3.8 to 4.3	
Psychologist	473	14.1	12.7 to 15.5	
Pharmacist	409	13.6	12.1 to 15.0	
Dentist	910	41.4	37.9 to 45.0	
Other health practitioner	474	9.1	8.2 to 9.9	
Age in 2010				< 0.0001
≤25	255	5.2	4.6 to 5.9	
26-35	1,334	8.0	7.5 to 8.5	
36-45	2,104	12.9	12.2 to 13.5	
46-55	2,594	16.4	15.7 to 17.2	
56-65	1,582	17.0	16.0 to 18.1	
≥66	438	16.4	14.4 to 18.3	
Sex				< 0.0001
Female	2,938	7.9	7.6 to 8.2	
Male	5,367	17.9	17.2 to 18.5	
Practice location				0.48
Major cities	6,343	12.5	12.1 to 12.9	
Inner/outer regional	1,840	12.0	11.3 to 12.7	
Remote/very remote	117	11.8	9.3 to 14.4	

(a) Some cells do not sum to 8,307 notifications because of missing data

(b) Adjusted for all other variables in the table and state/territory

(c) *p*-value refers to evidence that the adjusted notification rates differs between categories. This test is based on the coefficients (and their standard errors) from the negative binomial model

The error had no effect on the statistical significance of any values presented, nor did it affect other results reported in the paper.

Further to the above, a list of corrections to the main body relating to the errors in the original Figure 2 is located below:

- **Abstract**
  **Results**: There were 8307 notifications. The notification rate was highest among doctors (IR = 29.0 per 1000 practitioner years) and dentists (IR = 41.4) and lowest among nurses and midwives (IR = 4.1).
- **Results**
**Notification rates**
  In 2011–2012, 8307 notifications pertaining to 6920 practitioners were lodged with AHPRA. The overall rate was 12.7 notifications per 1000 practitioner years (95% CI, 12.4 to 12.9).
  Notification rates differed by profession, age, sex, and jurisdiction (Table 2). After adjusting for all of the variables shown in Table 2 plus jurisdiction, dentists had the highest rate of notifications (41.4 per 1000 practitioner years), followed by doctors (29.0 per 1000 practitioner years). Nurses and midwives had the lowest rate of notifications (4.1 per 1000 practitioner years). Risk of notification generally increased with age – practitioners aged ≤ 25 years were at lowest risk (5.2 per 1000 practitioner years) and practitioners aged 56–65 years were at highest risk (16.4 per 1000 practitioner years). Men were at much higher risk of notification than women (17.9 vs. 7.9 per 1000 practitioner years). Notification rates did not differ by remoteness of practice location (*P* = 0.48), but did by jurisdiction (*P* < 0.0001).
- **Discussion**
**Main findings**
  This study of notifications lodged over a 2-year period against practitioners from 10 health professions found an overall rate of 13 notifications per 1000 practitioner years.

Finally, the authors would like to note that in Reference 31 (see reference [2] here), the author ‘Patterson’ should instead be displayed as ‘Paterson’.
